# Silibinin Induces G2/M Cell Cycle Arrest by Activating Drp1-Dependent Mitochondrial Fission in Cervical Cancer

**DOI:** 10.3389/fphar.2020.00271

**Published:** 2020-03-12

**Authors:** Yanting You, Qiuxing He, Hanqi Lu, Xinghong Zhou, Liqian Chen, Huaxi Liu, Zibin Lu, Dongyi Liu, Yanyan Liu, Daming Zuo, Xiuqiong Fu, Hiuyee Kwan, Xiaoshan Zhao

**Affiliations:** ^1^School of Traditional Chinese Medicine, Southern Medical University, Guangzhou, China; ^2^Syndrome Laboratory of Integrated Chinese and Western Medicine, School of Traditional Chinese Medicine, Southern Medical University, Guangzhou, China; ^3^Traditional Chinese Pharmacological Laboratory, School of Traditional Chinese Medicine, Southern Medical University, Guangzhou, China; ^4^Department of Immunology, School of Basic Medical Sciences, Southern Medical University, Guangzhou, China; ^5^School of Chinese Medicine, Hong Kong Baptist University, Hong Kong, China

**Keywords:** silibinin, cervical cancer, G2/M cell cycle arrest, mitochondria fission, dynamin-related protein 1

## Abstract

Cervical cancer is the fourth leading cancer type and the second most common gynecological malignancy among women worldwide. Silibinin (SB), a chief bioactive natural polyphenolic flavonoid of *Silybum marianum* L., has been used clinically for its hepatocyte protective effects. It also has anticancer effects via the induction of apoptosis and cell cycle arrest. However, the effects of SB on cervical cancer cells through mitochondrial fission have not been studied. Here, we showed that SB markedly suppressed cervical cell proliferation by inducing G2/M cell cycle arrest via the activation of dynamin-related protein 1 (Drp1), which in turn mediated the mitochondrial fission dysfunction both *in vitro* and *in vivo*. SB decreased the ATP content, mitochondrial membrane potential, and mtDNA copy number, as well as reduced the reactive oxygen species levels in cervical cells. Furthermore, SB induced excessive mitochondrial fragmentation and reduced tubule formation. Further study showed that knockdown of Drp1 abolished the SB-induced G2/M cell cycle arrest in cervical cancer cells by inhibiting the mitochondrial fission pathway. More importantly, SB inhibited Hela cell growth *in vivo* model. In conclusion, we are the first to demonstrate that SB induces cervical cancer cell G2/M cell cycle arrest by activating Drp1-dependent mitochondrial fission dysfunction. This study suggests the strategy of inducing Drp1-dependent mitochondrial fission for cervical cancer prevention and treatment.

## Instruction

With an estimated 570,000 cases and 311,000 deaths in 2018 worldwide, cervical cancer is the fourth leading cancer type and the second most common gynecological malignancy among women throughout the world (Siegel et al., 2015). With high morbidity, more than half a million women are diagnosed as new cervical cancer patients annually (Bray et al., 2018). Heretofore, the standard therapeutic paradigms in cervical cancer are hysterectomy followed with chemotherapies, such as the 5-FU (Nayak et al., 2019). Although the treatments and diagnostic tools are improved, they have side effects such as refractory to platinum in many recurrent tumors (Cohen et al., 2019). Therefore, it is urgent to search for a cost-effective drug to treat cervical cancer.

With the increasing interest in screening for active anticancer compounds from natural products, a large number of the potential target-specific anti-cancer natural compounds have been identified recently (Dutta et al., 2019). With more than centuries of history of being used in folk medicine, *Silybum marianum* L. extract is known to have hepatocyte protective properties and it can prevent HCV infection. Therefore, it is commonly used in a clinical settings. Furthermore, *Silybum marianum* L. is a dietary supplement to reduce liver toxicity since three decades ago (Raina and Agarwal, 2007; Liu et al., 2017). Silibinin (SB) is one of the chief bioactive natural polyphenolic flavonoids isolated from the fruits and seeds of *Silybum marianum* L. (Zhang et al., 2015). SB is a well-tolerated hepatic protection medicine with minimal adverse effects (Ge et al., 2011). Moreover, SB has shown anti-cancer activity in several clinical treatments of tumors, such as in non-small cell lung cancer, prostate cancer, and colorectal cancer (Flaig et al., 2007; Siegel et al., 2014). There has not shown any apparent signs of toxicity or adverse effects in various acute and chronic animal tests (Raina and Agarwal, 2007). Given the traditional use of SB in the clinic and the high anti-cancer efficacy of SB, the mechanism underlying its anti-cervical cancer activity deserves further investigation (Bosch-Barrera et al., 2017).

The previous study has shown that SB induces breast cancer cell apoptosis due to mitochondrial dynamic impairment (Si et al., 2019). Mitochondrial dynamics contain network connection, the structure of mitochondria is with continuous fission (Westermann, 2008; Youle and van der Bliek, 2012). Fission is crucial to maintain the mitochondrial function and the cellular physiological, such as cell proliferation, production of energy, and cell cycle (Horbay and Bilyy, 2016; Kitamura et al., 2017; Rana et al., 2017). Cyclins and Cyclin-dependent kinases (CDKs) stringently regulate the cell cycle. Cyclin B1/CDK1 complex that regulates the checkpoint of G2/M, is especially important to control the cell enter mitosis (Wang et al., 2014). Accumulating evidence showed that the fission-mediating GTPase dynamin-related protein 1 (Drp1) could thoroughly change the mitochondrial fission by regulating cell cycling. Drp1 is activated by G2/M arrest and consequently induces the mitochondrial fission (Tanwar et al., 2016). Studies showed that the increased expression of Drp1 in mitochondrial fission could induce cervical cancer cell apoptosis (Bhushan et al., 2009).

In this study, we are the first to demonstrate that SB induces G2/M cell cycle arrest in cervical cancer cells via activation of the Drp1-mediates mitochondrial fission pathway. Our research has shown that SB is a promising medicine for the treatment of cervical cancer.

## Materials and Methods

### Cell Line and Culture

Persistent infection with high-risk human papillomavirus (HPV) has been verified as the major risk factor in cervical cancer progression (Liu et al., 2018). More than 118 different types of HPV has been validated and the type of HPV 16 and 18 attributed to three quarters incidence rates (Crosbie et al., 2013). So we chose the type of human cervical carcinoma cell lines Hela and SiHa cells, which infected by HPV 16, 18, and HPV 16, separately. Hela and SiHa cells (Guangzhou Cellcook Biotech Co., Ltd, China) were cultured in Dulbecco’s Modified Eagle medium (high glucose) supplemented with 10% fetal bovine serum (BI, Israel), penicillin (100 U/mL, BI, Israel) and streptomycin (100 μg/mL, BI, Israel), and maintained at 37°C and 5% CO_2_ in a humid environment. Cells in the mid-log phase were used in subsequent experiments.

### Cell Viability and Cell Growth Assay

Cell viability effects of SB (Chengdu Must Bio-Technology Co., Ltd, Chengdu, China, purity of SB is 98.89% identified by HPLC) were determined using 3-(4,5-dimethylthiazol-2-yl)-2,5-diphenylterazolium bromide (MTT). 5-FU is a commonly used chemotherapy drug in the treatment of cervical cancer (Nayak et al., 2019). SiHa (3 × 10^3^ cells/well) cells and Hela (3 × 10^3^ cells/well) were seeded onto 96-well microplate and cultured for 24 h, and then treated with SB or 5-FU (Sigma, United States, purity of 5-FU is ≥ 99%) at indicated concentrations for indicated periods. The cellular viability was assessed using MTT assay and was expressed as a ratio to the absorbance value at 570 nm of the control cells by a microplate reader (Multiskan FC, Thermo Fisher Scientific, Waltham, MA, United States).

### Colony Formation Assay

SiHa cells (150 cells/well) and Hela cells (150 cells/well) were seeded in 6-well plates and treated with SB for 24 h. Then, cells were washed with phosphate-buffered saline (PBS), and cultured in fresh medium for 15 days. After incubation, cells were fixed in 75% alcohol at 4°C overnight and stained with crystal violet dye for 30 min.

### Cell Proliferation Assays

KeyFluor488 Click-iT EdU kit (KeyGen BioTECH, Nanjing, China) was used to detect morphological changes that occurred after cells were treated with SB. Nuclear was stained with Hoechst 33342 (1 μg/mL) for 15 min at room temperature (RT). After washing by PBS, samples were visualized at 40× magnification (Olympus IX53, Tokyo, Japan).

### Flow Cytometry of Cell Cycle and Apoptosis

Cell cycle and apoptosis were measured by Cell Cycle Detection Kit and Annexin V-FITC Reagent separately (KeyGen BioTECH, Nanjing, China). Cells were harvested after 24 h SB treatment. For cell cycle analysis, cells were washed with PBS twice and fixed with 70% ethanol at 4°C overnight. PBS washed cells twice and incubated with RNase A for 30 min, then stained with PI at darkroom. For apoptosis, cells were washed with PBS twice, suspended and incubated with Annexin V-FITC and PI for 15 min at RT in the dark. Cell cycle and apoptosis were analyzed by flow cytometry (CytoFLEX, Backman Counter, CA, United States).

### Determination of Relative mtDNA Copy Number

Total DNA was extracted by Trizol reagent (Invitrogen, United States) and qPCR analysis was used to determine the relative mtDNA copy number. The qPCR amplification reaction was performed via SYBR Green chemistry using LightCycler^®^ 96 Real-time PCR system (Roche, Basel, Switzerland). The mtDNA was synthesized and amplified according to the manufacturer’s instructions as described previously (You et al., 2017).

### Measurement of Mitochondrial Membrane Potential (MMP)

MMP was determined using Mitochondrial membrane potential assay kit with JC-1 (Beyotime Institute of Biotechnology, Haimen, China) as described by the manufacture’s instruction. For each sample, JC-1 reagent was added and incubated for 20 min at 37°C. Cells were washed with PBS twice and observed by a fluorescence microscopy (Olympus FV1000, Tokyo, Japan). Living cells exhibit red fluorescent while dead or dying cells exhibit green fluorescence.

### Measurement of Intracellular Reactive Oxygen Species (ROS) and ATP

The levels of intracellular ROS were determined using a ROS assay kit (Beyotime Institute of Biotechnology, Haimen, China). Cells were stained with fluorescence dye DCFH-DA (10 μM/L) for 20 min in a darkroom and detected with flow cytometry. Cellular ATP levels were measured using an ATP Assay Kit (Beyotime Institute of Biotechnology, Haimen, China). The assay is based on luciferase’s requirement (PerkinElmer, Waltham, MA, United States) for ATP in producing light. Luminescence was read and values were calculated based on an ATP standard curve (Chen et al., 2018).

### Drp1 siRNA Transfection

SiHa cells (3 × 10^5^ cells/well) and Hela cells (3 × 10^5^ cells/well) were seeded in 6-well plates for 24 h and transfected with Drp1 siRNA (100 nM) or negative control using Lipo6000^TM^ (Beyotime Institute of Biotechnology, Haimen, China). After transfection for 24 h, cells were incubated with or without SB for another 24 h. Cells were collected for cell cycle analysis or Western blotting, respectively (Supplementary Figure S2).

### Immunofluorescent Staining

Cells were fixed in 4% paraformaldehyde for 15 min and permeabilized with 0.1% Triton X-100 for 15 min, blocked with 5% BSA in PBS for 1 h, and incubated with MitoRed (KeyGEN BioTECH, Jiangsu, China) for 1 h in darkness at RT. Then, cells were incubated with 4′, 6-diamidino-2-phenylindole (DAPI) for 5 min in darkness at RT. Samples were washed with PBS twice and imaged under a confocal microscope (LSM800, Carl Zeiss, Oberkochen, Germany).

### RNA Extraction and qPCR

After 24 h treatment of SB, cells were collected. Total cellular RNA was extracted from cells using the Trizol reagent and then subjected to qPCR analysis by SYBR^®^ Premix Ex TaqTM II (Tli RNaseH Plus). β*-actin* was used as internal controls.

### Western Blotting

The antibodies against Drp1, FIS1, cyclin B1, and β-actin were purchased from Affinity Biosciences (United States). CDK1 and cdc25C were purchased from Cell Signaling Technology (Danvers, MA, United States). For western blotting, cervical cancer cells were harvested and lysed by RIPA buffer for 30 min, then centrifuged at 12,000 × g for 15 min and the supernatant was collected. The proteins were quantified by BCA Protein assay kit (Thermo Fisher Scientific, Inc.). The membranes were then incubated overnight with anti-Drp1 (1:500), anti-FIS1 (1:500), anti-cyclinB1 (1:500), anti-cdc25C (1:1,000), CDK1 (1:500) and anti-β-actin (1:1,000) at 4°C. The membranes were washed once with TBST buffer and incubated with secondary antibody (Thermo Fisher Scientific, Inc., 1:1000) at room temperature for 1 h. The proteins levels of Drp1, FIS1, cdc25C, CDK1, cyclin B1, and β-actin from the cervical cancer cells were measured by the FluorChem E^TM^ system (ProteinSimple, San Francisco, CA, United States).

### Zebrafish Xenografts

Zebrafish have emerged as a remarkable model system for understanding the link between aberrant developmental pathways and tumorigenesis (Kendall and Amatruda, 2016). Wild-type-AB line zebrafish were kindly provided and housed in the key laboratory of Zebrafish Modeling and Drug Screening for Human Diseases Institute at Southern Medical University (Guangzhou, China) with standard procedures of Institutional Animal Care and Use Committee (IACUC). Adult zebrafish were maintained at a constant temperature of 28.5°C with a 14/10 h light/dark cycle. We chose Hela cells, which was more prone to tumor formation *in vivo*, to construct the zebrafish xenograft model. Hela cells were labeled with Dil dye (2 μM, Yeasen, Shanghai, China) and approximately 300 cells were injected into yolk sac of 2 days of post fertilization embryos and incubated with indicated concentrations of SB for 24 h in 37°C incubator. Each group was 50 embryos of 2 days postfertilization zebrafish, each 10 embryos was in one well of 24-well plate. Zebrafish embryos was in randomized grouping by number all the subjects and divided the subjects with random selected by software into the control group and experiments. SB was mixed with egg water and added in well of 24-well plate. The cancer cell proliferation was determined by fluorescence microscopy (Olympus MVX10, Olympus, Japan).

### Nude Mice Xenograft Assay

Xenograft human cancer models can be readily engrafted into immune-deficient mice (Dutta et al., 2019). All animal experiments were conducted by the animal use guidelines from the Animal Care and Use Committee of the Guangzhou Institute of Sport Science (Permit No.GZTKSGNX-2016-1). Four week-old female BALB/c nude mice (16 ± 2 g) (Guangdong Laboratory Animal Center, Guangzhou, China) were housed in a specific pathogen-free environment with constant temperature (22–25°C) and humidity (40–50%). We chose Hela cells, which was more prone to tumor formation *in vivo*, to construct the nude mice xenograft model. Hela cells (1 × 10^6^) in the exponential growth phase were harvested and injected (100 μL per site) into the right flank of each mouse. Therapeutic experiments were started when the tumor reached about 100 mm^3^. The mice were allocated to receive vehicle [control group, *n* = 6, intragastric administration (ig)], 150 mg/kg SB (*n* = 6, ig), 300 mg/kg SB (*n* = 6, ig) and 10 mg/kg 5-FU (*n* = 6, ig) in the same volume of 0.2 mL once a days. Both same group mice were in one cage. The tumor size was measured using a slide caliper, and the tumor volume = 0.5 × length × width^2^. Tumor volume was measured every 3 days. In nude mice xenografts, the tumor size was ≤ 600 mm^3^. The mice were euthanized by cervical dislocation and tumor tissues were excised and weighed.

### Statistical Analysis

All data are expressed as the means ± SDs and analyzed by SPSS 20.0 (IBM, Armonk, United States). Tukey’s test was used for multiple comparisons. The values were considered statistically significant when *P* < 0.05.

## Results

### SB Inhibits Cervical Cancer Cell Proliferation *in vitro*

**FIGURE 1:**
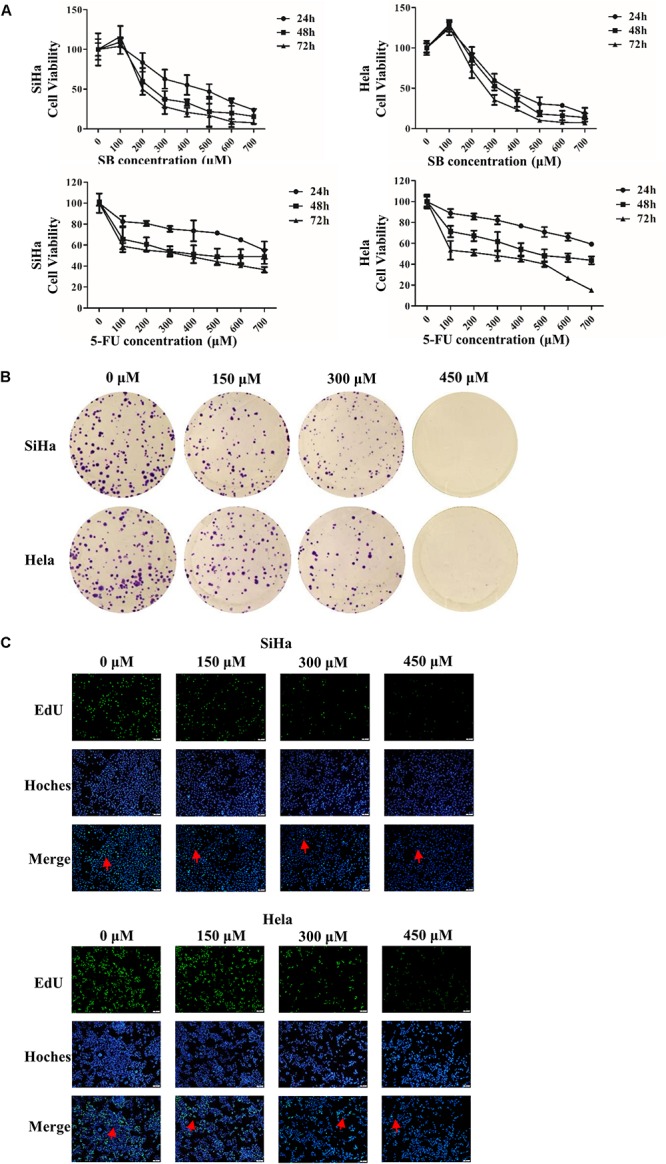
SB inhibits cervical cancer cell viability and growth. **(A)** Cells were exposed to silibinin (SB) (up panel) and 5-FU (down panel) at different concentrations for 24, 48, and 72 h, and cell viability was measured by MTT assays. **(B)** SB inhibited the proliferation of cervical cancer cells. **(C)** Cervical cancer cell proliferation was detected by keyFluor488 Click-iT EdU kit. Each drug dose point was repeated at least three independent experiments. Values (mean ± SDs) were obtained from at least three independent experiments.

We evaluated the effect of SB on the proliferation of cervical cancer cells. As shown in Figure 1A, SB inhibited the growth of both SiHa and Hela cells in a dose- and time-dependent manners, with IC_50_ values of 0.420, 0.250, and 0.195 mM in SiHa cells and 0.362, 0.332, and 0.275 mM in Hela cells at 24, 48, and 72 h, respectively. As the positive control, 5-FU substantially inhibited the cell growth in a dose- and time-dependent with IC_50_ values of 0.805, 0.523, and 0.211 mM in SiHa cells and 0.847, 0.489, and 0.376 mM in Hela cells at 24, 48, and 72 h, respectively, which showed that SB inhibited the proliferation of cervical cancer cell slightly higher than 5-FU *in vitro*. However, there was no significant inhibition in normal human renal mesangial cells treated with SB (Supplementary Figure S1). We further detected the proliferation ability of cervical cancer cells treated with SB by the EdU incorporation assays and colony formation assays (Figures 1B,C). The results confirmed that SB markedly suppressed the proliferation and colony formation of cervical cancer cells dose-dependently.

### Effect of SB on the Induction of Apoptosis in Cervical Cancer Cells

**FIGURE 2:**
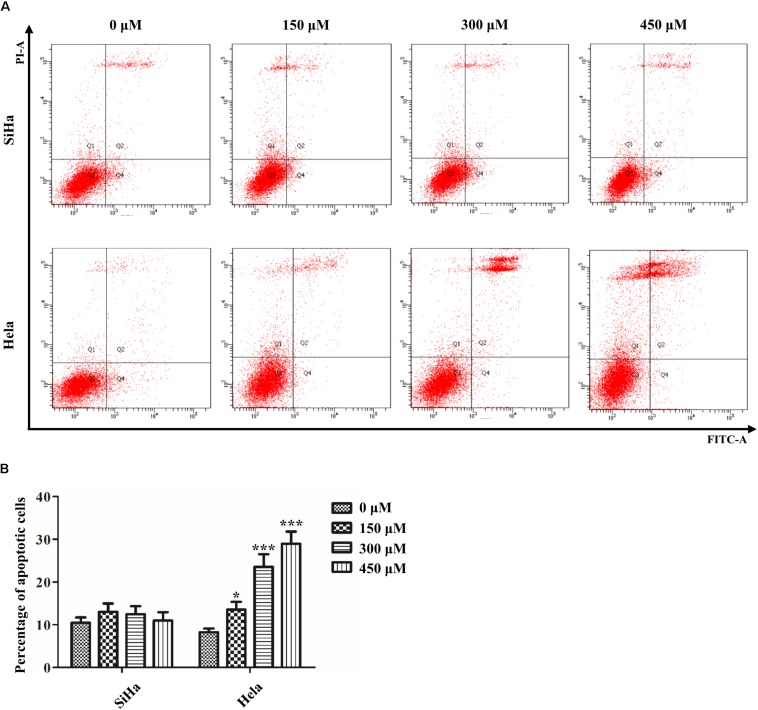
Effect of SB on the induction of apoptosis in cervical cancer cells. **(A)** A marked dose-dependent increase of apoptosis for Hela cells by flow cytometry. **(B)** No significant change of apoptosis for SiHa cells by flow cytometry. Values (mean ± SDs) were obtained from at least three independent experiments. *P < 0.05 and ***P < 0.001 by one-way ANOVA with Tukey’s test.

Next, we further determined whether the suppressive effect of SB on the growth of cervical cancer cells was associated with the induction of apoptosis. Annexin V-FITC/PI staining assay showed that SB induced Hela cells apoptosis in a dose-dependent manner (Figures 2A,B). However, SB did not induce apoptosis in SiHa cells with all the tested concentrations. The different impact of SB on Hela and SiHa cell apoptosis indicated that the underlying mechanism of SB in suppressing cell growth is distinct in different cervical cancer cells.

### SB Induces G2/M Cell Cycle Arrest in Cervical Cancer Cells

**FIGURE 3:**
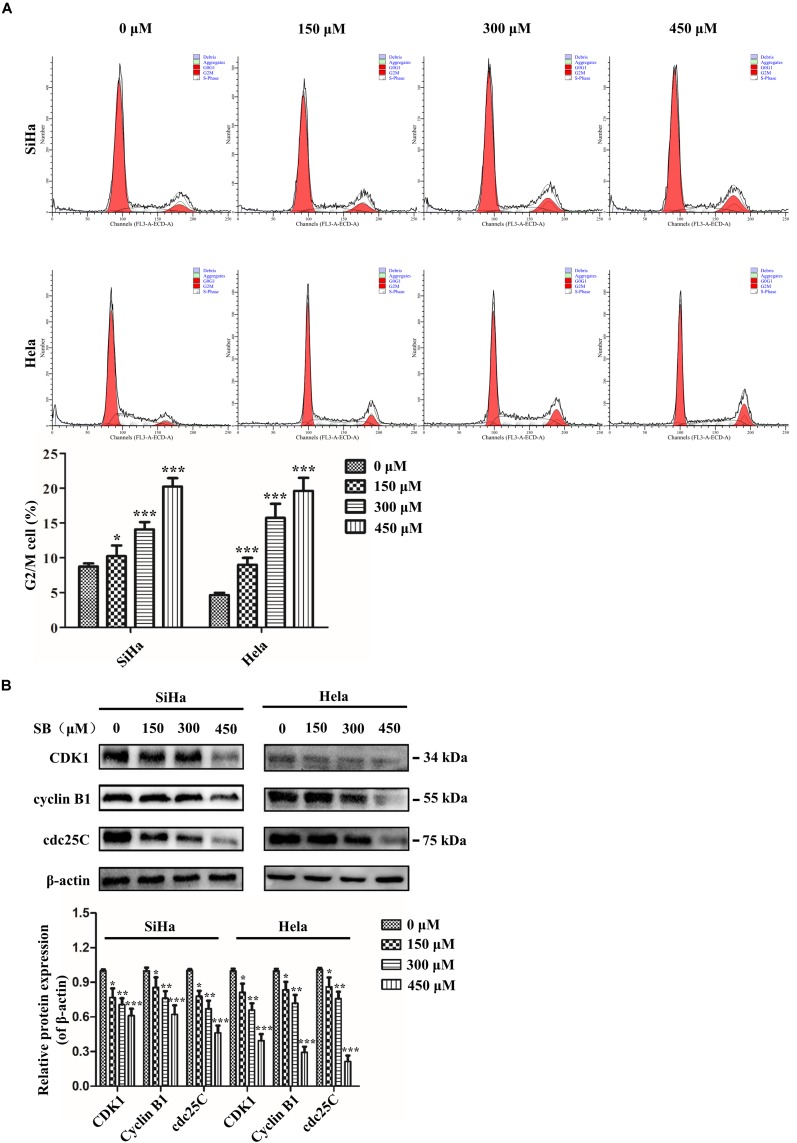
SB induces G2/M cell cycle arrest in cervical cancer cells. **(A)** A marked dose-dependent increase of the percentage of cervical cancer cells in the G2/M phase arrest by flow cytometry. **(B)** The protein expression levels of CDK1, cyclin B1, and cdc25C at 24 h after SB treatment. Expression levels were normalized to the β-actin protein level. Values (mean ± SDs) were obtained from at least three independent experiments. *P < 0.05, **P < 0.01, and ***P < 0.001 by one-way ANOVA with Tukey’s test.

It is well known that uncontrolled cell proliferation is due to the disorder of the cell cycle, which is a characteristic of cancer. To demonstrate the potential regulating role of SB on the cell cycle, we analyzed the different phases of the cell cycle by flow cytometry. The data showed that SB treatment led to a remarkable accumulation of both SiHa cells and Hela cells in the G2/M phase arrest in a dose-dependent manner (Figure 3A), suggesting that SB induces G2/M cell cycle arrest in cervical cancer cells.

To further explore the mechanisms underlying the SB-induced G2/M cell cycle arrest, we examined the cell cycle-associated regulatory proteins for G2/M transition (CDK1, cyclin B1, and cdc25C) by western blotting. The results showed that SB down-regulated the expressions of CDK1, cyclin B1, and cdc25C at protein levels (Figure 3B), which further suggests that SB induces G2/M cell cycle arrest in cervical cancer cells.

### SB Impairs Mitochondrial Fission in Cervical Cancer Cells

**FIGURE 4:**
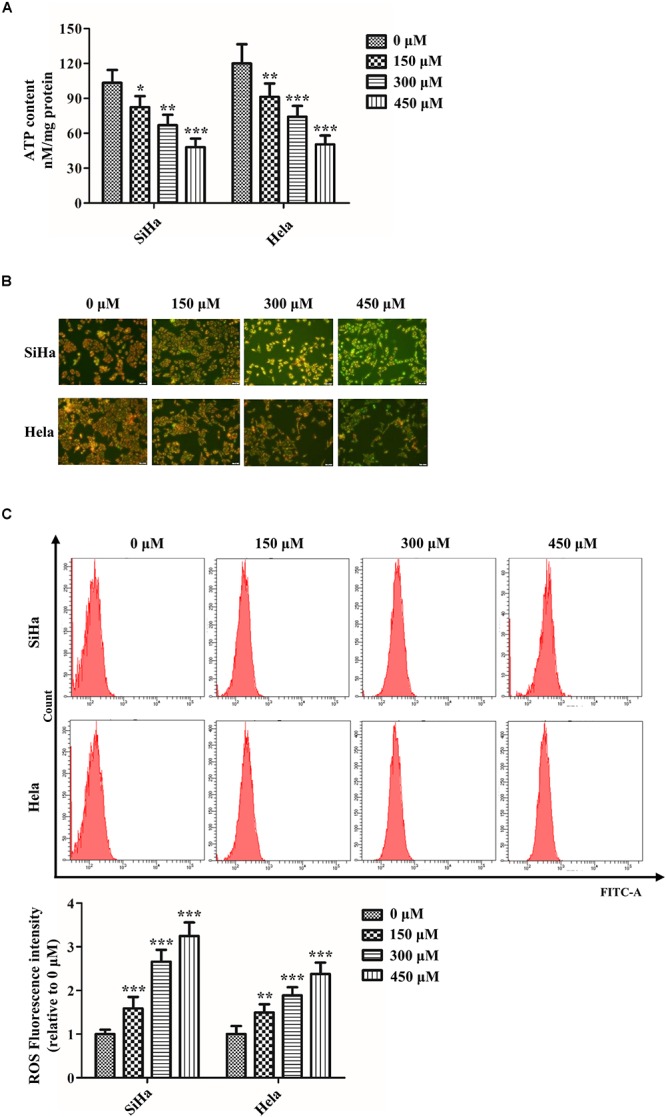
SB impaired mitochondrial dynamics in cervical cancer cells. **(A)** Content of ATP in cervical cancer cells detected by the luminometric assay. **(B)** Effect of SB on mitochondrial membrane potential (MMP) change in SB-induced mitochondrial dysfunction. **(C)** Promotion of reactive oxygen species (ROS) levels in cervical cancer cells treated by SB detected by flow cytometry. Values (mean ± SDs) were obtained from at least three independent experiments. *P < 0.05, **P < 0.01, and ***P < 0.001 by one-way ANOVA with Tukey’s test.

**FIGURE 5:**
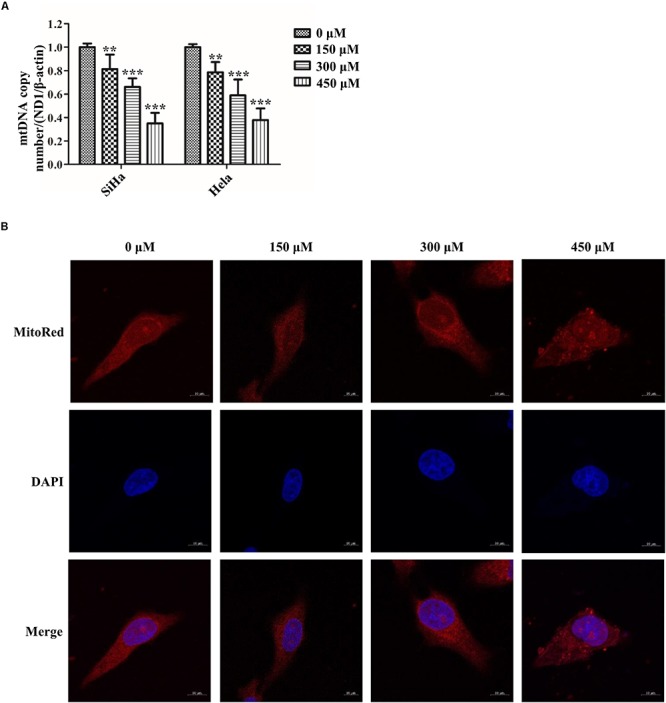
SB impaired mitochondrial fission in cervical cancer cells. **(A)** The expression of mitochondrial mtDNA copy number determined by quantitative polymerase chain reaction (qPCR). **(B)** Hela cells mitochondrial morphology treated by SB for 24 h were stained with MitoRed (red) and co-stained with DAPI (blue) to demonstrate mitochondrial morphology (red) and nucleus (blue) by fluorescence microscopy, respectively. Values (mean ± SDs) were obtained from at least three independent experiments. ^∗∗^*P* < 0.01 and ^∗∗∗^*P* < 0.001 by one-way ANOVA with Tukey’s test.

Abnormal mitochondrial fission plays a pivotal role in cancer metabolism and transition. To investigate the influence of SB in mitochondrial fission, the mitochondrial biogenesis was evaluated. The primary function of mitochondria is to generate energy in the form of ATP. Our results revealed that SB inhibited the mitochondrial ATP production dose-dependently in cervical cancer cells (Figure 4A). MMP, closely related to mitochondrial ATP content, is a distinctive characteristic of mitochondrial fission. By using the JC-1 probe, we found that SB reduced the MMP (red fluorescence) expression in cervical cancer cells (Figure 4B). Additionally, SB treatment markedly increased the levels of mitochondrial ROS in cervical cancer cells (Figure 4C). MtDNA has closely related to cell respiratory and the mtDNA copy number reduction is the main consequence of oxidative stress. The results showed that the mtDNA copy number was dramatically reduced in a dose-dependent manner upon SB treatment (Figure 5A). These results suggest that SB promotes oxidative damage to the mitochondria in cervical cancer cells.

**FIGURE 6:**
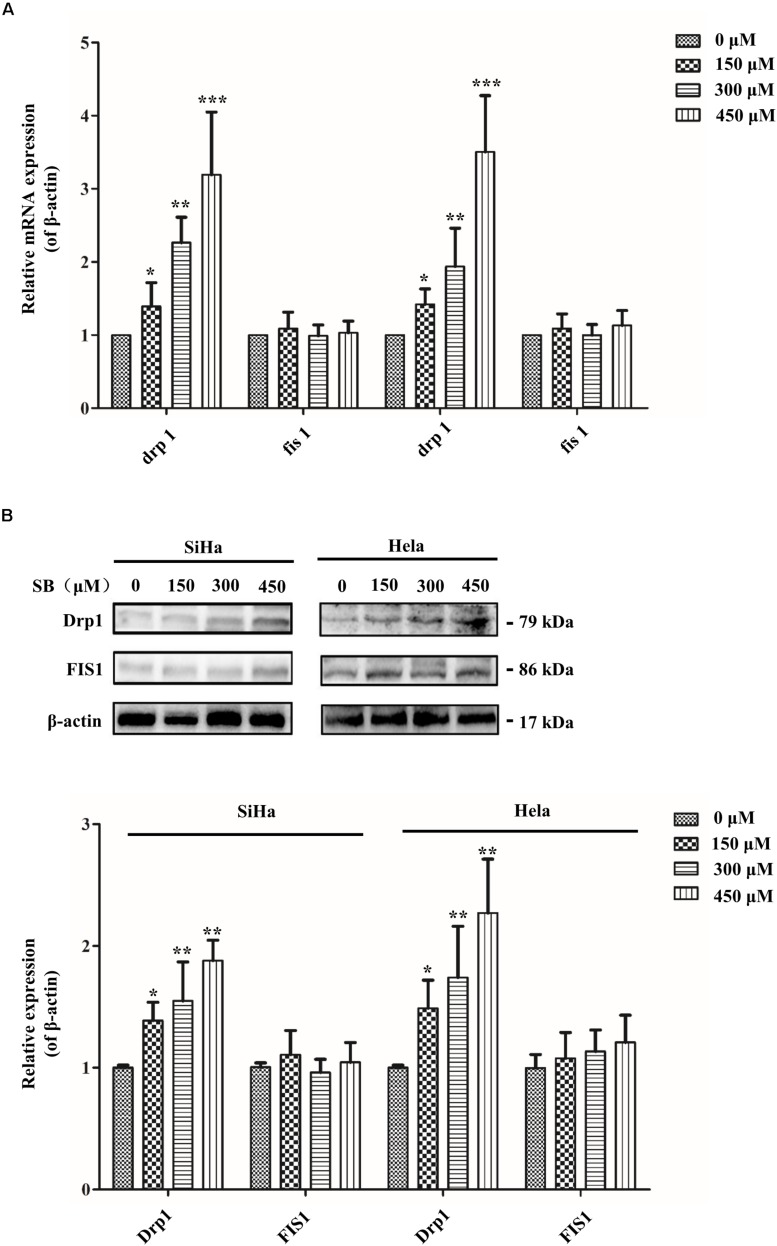
SB impaired mitochondrial fission in cervical cancer cells. **(A)** The expression of Drp1 significantly increased in a dose-independent manner in cervical cancer cells. Mitochondrial fission related mRNA levels were normalized to β-actin gene levels. **(B)** The mitochondrial fission related protein expression extracted from cervical cancer cells. Drp1 expression was increased in both two cervical cancer cells. The mitochondrial fission related protein and gene expression level in cervical cancer cells were detected by western blotting and qPCR, respectively. Values (mean ± SDs) were obtained from at least three independent experiments. *P < 0.05, **P < 0.01, and ***P < 0.001 by one-way ANOVA with Tukey’s test.

To meet the cellular demands of cancer cells, cellular biogenesis is mediated through the mitochondrial fission (Xie et al., 2015). Therefore, whether SB active mitochondrial fission was been studied. MitoRed staining was used to detect the mitochondrial morphology. As shown in Figure 5B, SB generated excessive mitochondrial fragmentation and reduced tubules formation in cervical cancer cells. Researchers have demonstrated that Drp1 is a key regulator of the mitochondrial fission process. To test whether the Drp1 was involved in the SB-mediated mitochondrial fission disorder in cervical cancer cells, q-PCR and western blotting were used to examine the gene and protein levels of Drp1, respectively. SB treatment substantially increased the expression of Drp1 in a dose-dependent manner, but did not influence the expression of FIS1 in cervical cells after 24 h SB treatment (Figures 6A,B). Collectively, these results indicate that SB induces mitochondrial fission dysfunction in cervical cancer cells, which might be associated with the Drp1-mediates mitochondrial fission pathway.

### SB Induces G2/M Cell Cycle Arrest in Cervical Cancer Cells by Activating the Drp1-Mediates Mitochondrial Fission Pathway

**FIGURE 7:**
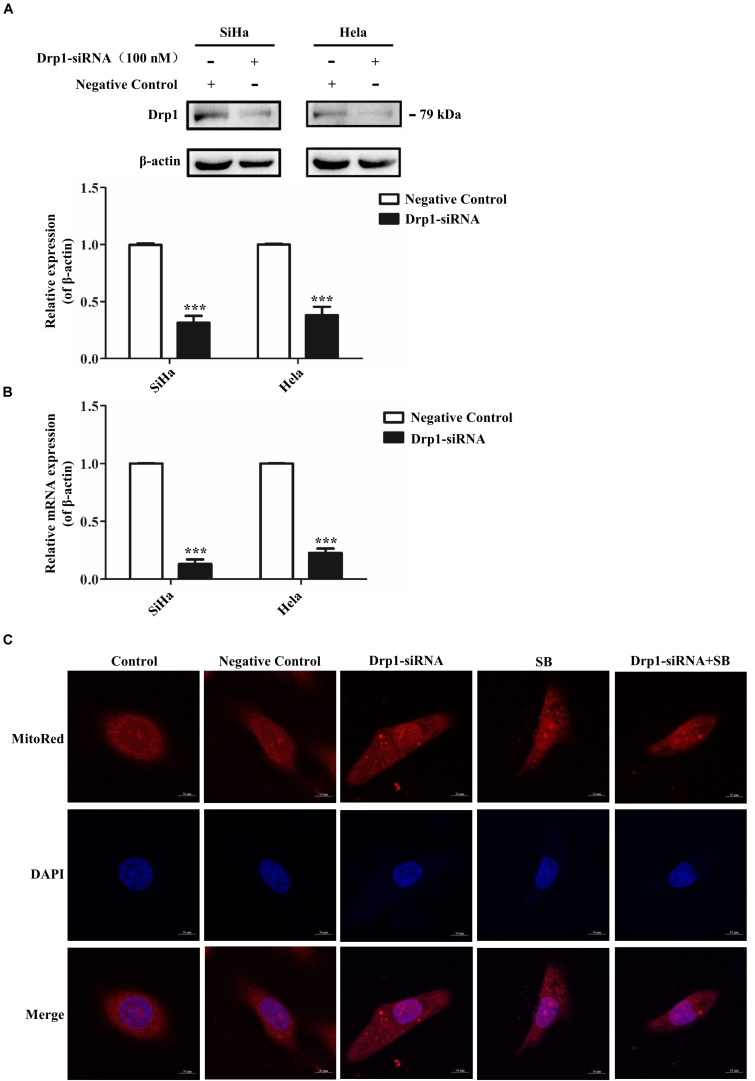
SB induces mitochondrial dynamic dysfunction in cervical cancer cells by activating the Drp1 mediated mitochondrial fission pathway. **(A,B)** The functional verification of Drp1-siRNA. The protein and gene expression levels of Drp1 in cervical cancer cells were detected by western blotting **(A)** and qPCR **(B)**, respectively. **(C)** Blockage of Drp1 relieved SB-induced mitochondrial tubules elongation. Hela cells mitochondrial morphology were stained with MitoRed (red) and co-stained with DAPI (blue) to demonstrate mitochondrial morphology (red) and nucleus (blue) by fluorescence microscopy, respectively. Values (mean ± SDs) were obtained from at least three independent experiments. ***P < 0.001 by one-way ANOVA with Tukey’s test.

Drp1 is the key factor in the regulation of mitochondrial fission and fusion during cell cycle progression (Yamano and Youle, 2011). To further suggest the role of Drp1 SB-induced cell cycle arrest, Drp1-specific siRNA was used to knockdown Drp1 expression in cervical cancer cells. As shown in Figures 7A,B, siRNA targeted to Drp1 efficiently reduced the expression of Drp1 in cervical cancer cells. As expected, knockdown of Drp1 significantly increased the elongations of mitochondrial tubules. Notably, Drp1 deficiency abrogated the SB induced mitochondrial dynamic dysfunction (Figure 7C). These data suggest that SB induces mitochondrial dynamic dysfunction in cervical cancer cells through the activation of the Drp1 mediated mitochondrial fission pathway.

**FIGURE 8:**
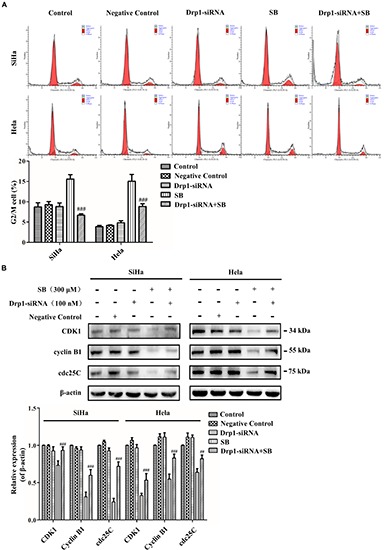
SB induces mitochondrial dynamic dysfunction and G2/M cell cycle arrest in cervical cancer cells by activating the Drp1 mediated mitochondrial fission pathway. **(A)** The blockage of Drp1 decreased the G2/M cell cycle arrest. The cell cycle was analyzed by flow cytometry. **(B)** Inhibition of Drp1 increases the change of G2/M cell cycle-related proteins. The expression level of CDK1, cyclin B1 and cdc25C were determined by western blotting. Values (mean ± SDs) were obtained from at least three independent experiments. ^##^*P* < 0.01 and ^###^*P* < 0.001, versus SB treatment group by one-way ANOVA with Tukey’s test.

Cyclin B1/CDK1 complex is a primary protein kinase in mitochondrial dynamics and stimulates mitochondrial fission activity (Taguchi et al., 2007). To further suggest the effect of Drp1in mediating mitochondrial fission in SB-induced cell cycle arrest, Drp1-siRNA was used to knockdown Drp1. Our results showed that Drp1-knockdown in cervical cancer cells reduced the G2/M cell cycle arrest (Figure 8A) and reversed the reduction of CDK1, cyclin B1 and cdc25C expressions compared to control cells upon SB treatment (Figure 8B). These results demonstrate that SB induces G2/M cell cycle arrest in cervical cancer cells by activating the Drp1-mediates mitochondrial fission pathway.

### SB Inhibits the Growth of Hela Cells in the Tumor Model

**FIGURE 9:**
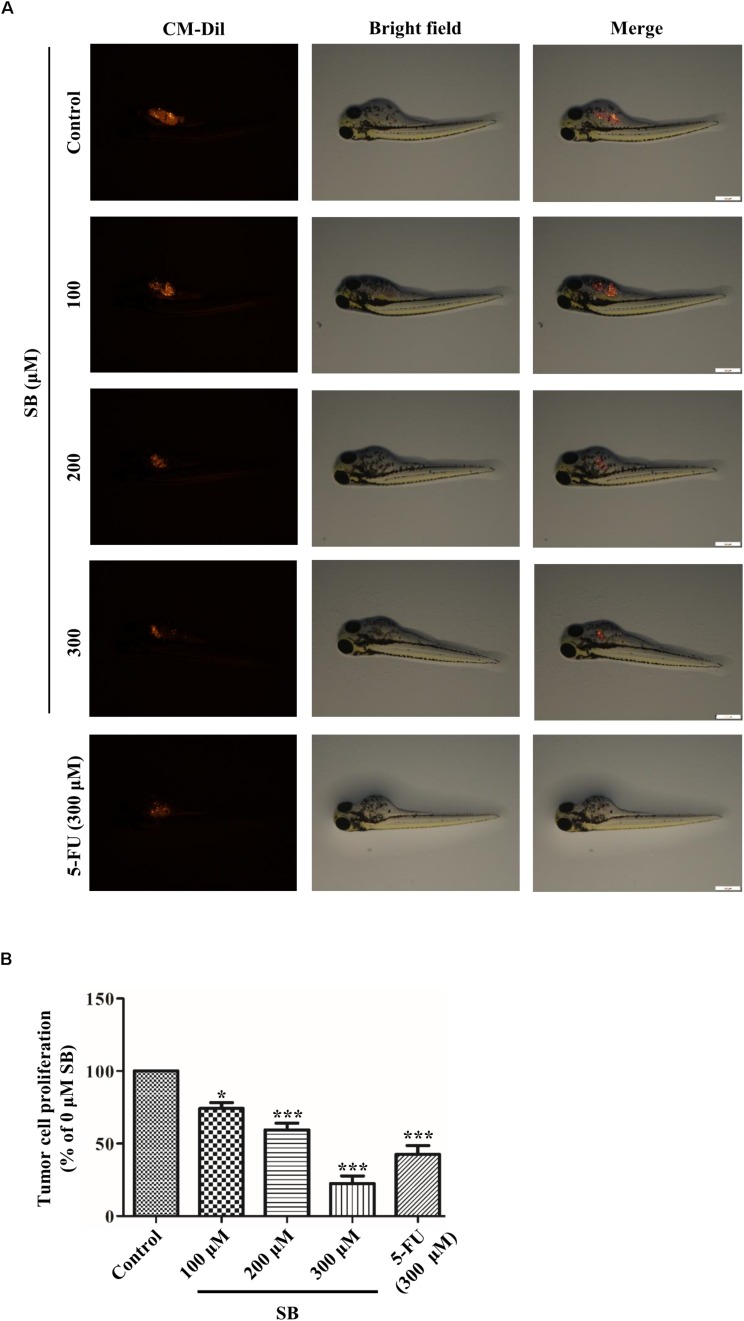
SB inhibits the growth of Hela cells in the zebrafish tumor model. In the zebrafish tumor model, anti- cervical cancer abilities of SB and 5-FU were evaluated by CM-Dil staining. Approximately 300 Dil-labeled Hela cells were injected into the yolk sac of each zebrafish and monitored and evaluated by the red fluorescence intensity **(A,B)**. Values (mean ± SDs) were obtained from independent experiments. *P < 0.05 and ***P < 0.001, versus control group by one-way ANOVA with Tukey’s test.

**FIGURE 10:**
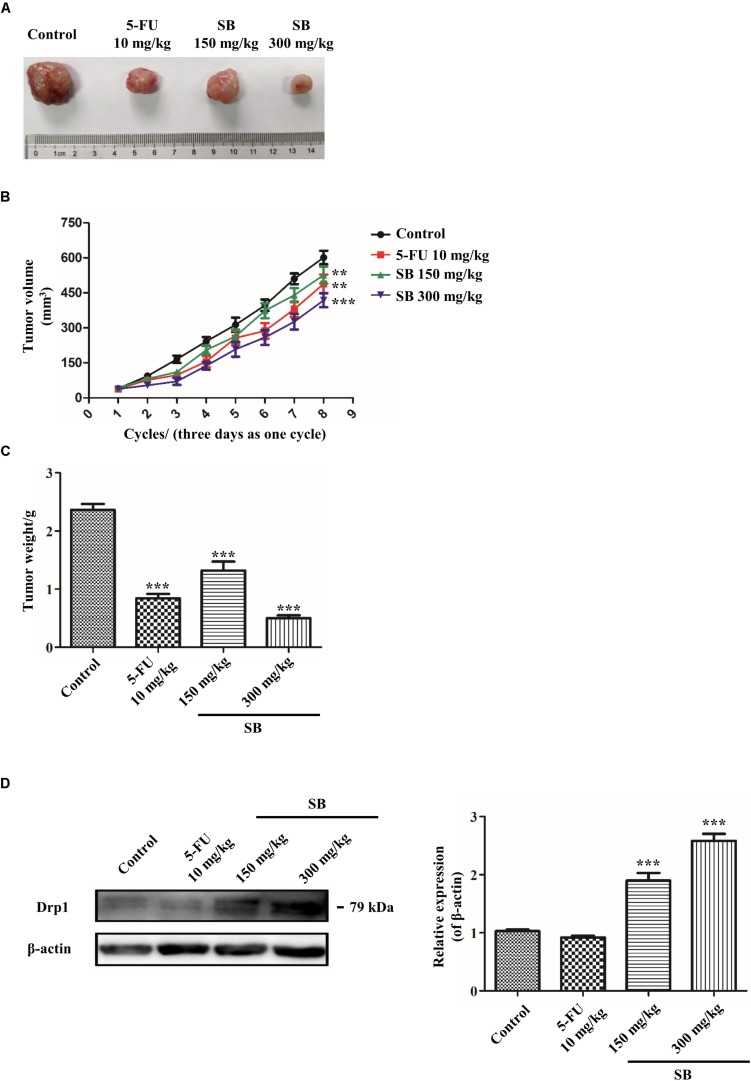
SB inhibits the growth of Hela cells in the xenograft mouse model. Therapeutic experiments were started when the tumor reached about 100 mm^3^. The tumor volume was measured every 3 days. **(A)** The photographs of dissected tumors from xenograft mice were shown. **(B,C)** The growth curves and the average weights if tumors from xenograft mice. **(D)** The Drp1 protein expression extracted from cervical cancer nude mice tumor tissue. The Drp1 protein expression level was detected by western blotting. Values (mean ± SDs) were obtained from independent experiments. **P < 0.01 and ***P < 0.001, versus control group by one-way ANOVA with Tukey’s test.

To determine whether SB exhibited antitumor activity *in vivo*, the zebrafish tumor model and xenograft mouse model were established. Zebrafish have emerged as a remarkable model system for understanding the link between aberrant developmental pathways and tumorigenesis (Kendall and Amatruda, 2016). We investigated whether SB affected Hela cells *in vivo* by establishing a zebrafish tumor model with cell membrane dye Dil. Approximately 300 Dil-labeled Hela cells were injected into the yolk sac of each zebrafish. The red fluorescence intensity of Dil dye was monitored and evaluated by a fluorescence microscopy. After treating with SB, the red fluorescence intensity was decreased in a dose-dependent manner, suggests that SB reduces the Hela cells number, and the number of Hela cell was significantly higher in 5-FU than SB treatment at the same concentration in the zebrafish model (Figures 9A,B). We further assessed whether SB exerted an anti-tumorigenic effect in the murine model. Hela cells were subcutaneously transplanted into nude mice. SB treatment significantly inhibited tumor growth in a dose-dependent manner. Compared with 5-FU treatment, tumor growth could be significantly inhibited at the high concentration of SB (Figures 10A–C). During the experiment, the status of the animal all in good condition (Supplementary Figure S3) To test whether the Drp1 was involved in the SB-mediated mitochondrial fission disorder in a cervical cancer tumor model, western blotting was used to examine the protein levels of Drp1. SB treatment substantially increased the expression of Drp1 in a dose-dependent manner in nude mice tumor tissues after SB treatment (Figure 10D). Together, *in vivo* data indicates that SB inhibited tumor growth in both the zebrafish tumor model and the xenograft mouse model.

## Discussion

SB is a major bioactive polyphenolic flavonoid isolated from the seed of the *Silybum marianum L*. Recently, studies reported that SB possess various potential biological effects, especially its remarkable anti-cancer effect in cancer by inducing cell cycle and apoptosis (Deep and Agarwal, 2010; Zeng et al., 2011; Bosch-Barrera and Menendez, 2015). However, the mechanism underlying the SB-induced apoptosis and cell cycle arrest in Hela cells are less studied. In this study, we are the first to demonstrate that SB decreases cell proliferation in both Hela and SiHa cells in a dose- and time-dependent manner. Moreover, it is worth noting that, SB inhibited the proliferation of Hela and SiHa cells, but did not induce apoptosis in SiHa cells, suggesting that SB inhibits SiHa cell proliferation is not dependent on apoptosis. Mechanistically, we found that SB treatment induced G2/M cell cycle arrest via the activation of Drp1 which mediated mitochondrial dynamic dysfunction. This study suggests the role of Drp1 in mitochondrial fission.

Apoptosis, a programmed cell death, plays a crucial role in both cancer development and drug responsiveness. Extrinsic death receptor pathway and mitochondrial pathway are the two major pathways in the execution of apoptosis (Khanam et al., 2018). In this study, we found that SB induced Hela cells apoptosis in a dose-dependent manner. However, SB did not induce apoptosis in SiHa cells with all the tested concentrations. The different impact of SB on Hela and SiHa cell apoptosis indicated that the underlying mechanism of SB in suppressing cell growth is distinct in different cervical cancer cells. The same mechanism of SB induced cervical cancer cells death was studied in this study. We will further investigate the mechanism of SB induced different death pathway in cervical cancer in future studies.

Aberrant cell cycle progression beyond the restriction point is the critical factor leading to the uncontrolled proliferation of cancer cells (Otto and Sicinski, 2017). In the progression of the cell cycle, members of the CDK family and the partner cyclin-proteins are the primary mediators controlling the point of entry into mitosis in cells (Jones et al., 2018). Cyclin B1/CDK1 kinase translocating rapidly from the cytoplasm to the nucleus is the critical target of G2 into mitosis transition (Giono et al., 2017). CDK1 promotes the DNA replicative synthesis and regulates the balance of cell cycle, thus coordinating and maintaining genomic stability, which is vital for cell survival (Liao et al., 2017). Precious studies found that CDK1-mediated regulation of cell proliferation could be modulated by regulatory subunit cyclin B1 (Jones et al., 2018). The cdc2 family (cdc25A, B, and C in mammals) carried out by the cyclin B1/CDK1 complex drive cell entry into mitosis. Cdc25C shuttles in and out of the nucleus continuously, just like cyclin B1. Cdc25C will not cause premature mitosis before cyclin B1 overexpress (Karlsson et al., 1999) and cdc25C is a member of the cyclin-dependent kinases of cell cycle-related CDKs (Giono et al., 2017). In the present study, we found that SB induced cell cycle arrest at G2/M phase and reduced the protein expressions of cdc25C, CDK1, and cyclin B1 in cervical cancer cells, which may be the mechanism of action underlying the SB-inhibited cervical cancer cell proliferation.

In the entire mitochondrial network structure, the mitochondrial fission is one of the key determinants of the crucial cellular bioenergetics including metabolism, cell cycle progression, proliferation and apoptotic cell death (Lackner, 2014). Mitochondrial fission has a critical function in energy production. Mitochondria produce ATP as energy to maintain cell metabolism, ROS generation, and balance of apoptosis and cell cycle (Nunnari and Suomalainen, 2012). Under the pathological conditions, the decreased production of ATP in cell will lead to mitochondrial fission dysfunction (Cho et al., 2010). Mitochondria is a major source of ROS. Excessive ROS will destroy the mitochondrial fission function (Hu et al., 2019). Increasing ROS production and electron leak induced by highly reactive oxygen, will disturb and lead to MMP reduction, which affects the function of mtDNA. Since the mitochondrial genome does not contain a non-coding sequence, mtDNA is highly susceptible to mutations by the increasing of ROS (Clerkin et al., 2008). In the present study, we found that SB treatment was able to reduce the generation of ATP and increase the excessive production of ROS in cervical cancer cells, which consequently induced MMP and reduced mtDNA copy number.

The function of mitochondrial fission is primarily controlled by exclusive GTPases. During mitochondrial fission, a mitochondrion divides into two smaller mitochondrion by fission of outer and inner membranes, which is regulated by Drp1, a large dynamic-related GTPase. Drp1 recruits mitochondria by specific mitochondria localized receptors FIS1 and MFF1 to forms an oligomeric loop in the mitochondrial outer membrane (Mishra and Chan, 2014; Jones et al., 2017). Here, we found that SB enhanced the expression of Drp1, whereas it did not affect the expression level of FIS1. Meanwhile, SB also led to excessive mitochondrial fragmentation and fewer tubules in cervical cancer cells. Based on these results, we hypothesize that SB causes cell cycle G2/M arrest by impairing mitochondrial dynamic in cervical cancer cells. The inhibition of cervical cancer cell proliferation induced by SB may due to the increased expression of Drp1, which reduced CDK1, cyclinB1, and cdc25C expressions in G2/M cell cycle arrest. Further studies are needed to determine the relationship between G2/M cell cycle arrest and Drp1 activation in cervical cancer cells after SB treatment.

**FIGURE 11:**
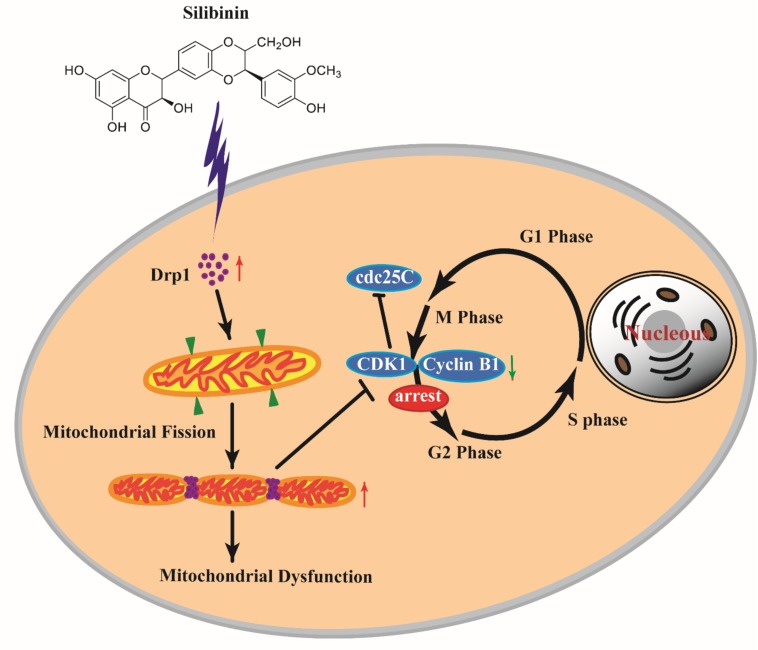
SB induced G2/M cell cycle arrest in cervical cancer cells through the mitochondrial fission dynamic pathway mediated by Drp1 *in vitro* and *in vivo*. SB increased the expression of Drp1 in cervical cancer cells which lead to mitochondrial excessive fission and mitochondrial dysfunction, thereby inducing the G2/M cell cycle arrest and decreasing the expressions of CDK1, cyclin B1, and cdc25C. Blocking Drp1 inactivates mitochondrial fission in cervical cancer cells and alleviates the G2/M cell cycle arrest. Our study suggests that SB can be exploited as a potential medicine for cervical cancer therapeutic prevention and treatment *via* the Drp1-mediated mitochondrial pathway.

Previous reports showed that the activity of Drp1 is modulated by G2/M cell cycle arrest (Tanwar et al., 2016). Mitochondrial morphology remodeling throughout the cell cycle is sufficient to meet cellular energy requirements at specific stages of the cell cycle and to ensure the faithful inheritance of mitochondria during cell division. Scattered mitochondrial network morphology promotes cell cycle arrest (Qian et al., 2012). The characteristics of SB on mitochondrial dysfunction in cancer have been reported, especially for SB-driven mitochondrial apoptotic pathway (Si et al., 2019; Yu et al., 2019). However, to our knowledge, there is no study examining the relationship between mitochondrial fission and cell cycle upon SB administration in cancer cells. To verify whether SB-induced G2/M cell cycle arrest is mediated by Drp1 in cervical cancer cells, we investigated if downregulation of Drp1 could reduce SB-induced cell G2/M cycle arrest. Meanwhile, Drp1 siRNA alleviated the mitochondrial fragmentation caused by SB and elongated mitochondrial tubules, indicating that the activity of Drp1 contributes to SB-induced mitochondrial fragmentation in the cervical cancer cell. These results further validate that mitochondrial fission/fusion dynamic plays a vital role in SB-mediated G2/M cell cycle arrest in cervical cancer cells (Figure 11).

In cancer research, cell transplantation approaches have been used to detect molecular pathways involved in tumor growth, metastasis, and anti-tumor therapies. Among these approaches, xenograft human cancer models can be readily engrafted into immune-deficient mice. Recently, zebrafish, a vertebrate model system, becomes an important new cancer model that complements the functions of the traditional xenograft mouse model and also has the capabilities to capture the heterogeneous and evolving complexity of cancer *in vivo* (White et al., 2013). We found that in both zebrafish tumor model and xenograft mouse model, SB inhibited Hela cells growth. Furthermore, SB was significantly increased the expression of Drp1 in cervical cancer tumor tissue of nude mice, which suggests the possibility of using SB as a new clinical therapeutics for cancer treatment. However, we only observed SB inhibited tumor growth for 24 h in the zebrafish xenograft, but we didn’t know whether SB has long- term effect for reducing tumor growth or whether the tumor will recur if removed SB treatment. Then we would like to use 5-FU combined with low concentration SB in the following research to see if SB could enhance the anti-tumor effect and relieve the adverse effect of 5-FU.

## Conclusion

Our study is the first to illustrate that SB induces G2/M cell cycle arrest in cervical cancer cells through the mitochondrial fission/fusion dynamic mediated by Drp1, and suggests a novel therapeutic strategy for cervical cancer prevention and management *via* the regulation of the mitochondrial pathway.

## Data Availability Statement

The raw data supporting the conclusions of this article will be made available by the authors, without undue reservation, to any qualified researcher.

## Ethics Statement

The animal study was reviewed and approved by the animal use guidelines from the Animal Care and Use Committee of the Guangzhou Institute of Sport Science (Permit No. GZTKSGNX-2016-1).

## Author Contributions

XZha conceived, designed, and supervised the study. YY, QH, LC, and HL performed cell research. YY, DL, and CZ performed the Zebrafish xenografts experiments. YY, HL, and XZho performed the nude mice xenograft assay. YY and QH did data analysis and interpretation. YY, QH, and HL wrote the manuscript. ZL, YL, DZ, XF, and HK modified the manuscript. All authors have read and approved the final manuscript.

## Conflict of Interest

The authors declare that the research was conducted in the absence of any commercial or financial relationships that could be construed as a potential conflict of interest.
