# Supplementary material for: Silibinin Induces G2/M Cell Cycle Arrest by Activating Drp1-Dependent Mitochondrial Fission in Cervical Cancer
Source: Front Pharmacol. 2020 Mar 12;11:271. doi: 10.3389/fphar.2020.00271 (PMC7080994; doi:10.3389/fphar.2020.00271)
Supplement: Supplementary file 1 [file Data_Sheet_1.PDF]

The ARRIVE Guidelines Checklist

Animal Research: Reporting In Vivo Experiments

Carol Kilkenny[1], William J Browne[2], Innes C Cuthill[3], Michael Emerson[4] and Douglas G Altman[5]

[1]The National Centre for the Replacement, Refinement and Reduction of Animals in Research, London, UK, [2]School of Veterinary Science, University of Bristol, Bristol, UK, [3]School of Biological Sciences, University of Bristol, Bristol, UK, [4]National Heart and Lung Institute, Imperial College London, UK, [5]Centre for Statistics in Medicine, University of Oxford, Oxford, UK.

	ITEM	RECOMMENDATION	Section/ Paragraph
Title	1	Silibinin induces G2/M cell cycle arrest by activating Drp1-dependent mitochondrial fission in cervical cancer	Title/1
Abstract	2	Cervical cancer is the fourth leading cancer type and the second most common gynecological malignancy among women worldwide. Silibinin (SB), a chief bioactive natural polyphenolic flavonoid of *Silybum marianum L.*, has been used clinically for its hepatocyte protective effects. It also has anticancer effects via the induction of apoptosis and cell cycle arrest. However, the effects of SB on cervical cancer cells through mitochondrial fission have not been studied. Here, we showed that SB markedly suppressed cervical cells proliferation by inducing G2/M cell cycle arrest via the activation of dynamin-related protein 1 (Drp1), which in turn mediated the mitochondrial fission dysfunction both *in vitro* and *in vivo*. SB decreased the ATP content, mitochondrial membrane potential and mtDNA copy number, as well as reduced the reactive oxygen species levels in cervical cells. Furthermore, SB induced excessive mitochondrial fragmentation and reduced tubule formation. Further study showed that knockdown of Drp1 abolished the SB-induced G2/M cell cycle arrest in cervical cancer cells by inhibiting the mitochondrial fission pathway. More importantly, SB inhibited Hela cell growth *in vivo* model. In conclusion, we are the first to demonstrate that SB induces	Abstract/1

		cervical cancer cell G2/M cell cycle arrest by activating Drp1-dependent mitochondrial fission dysfunction. This study suggests the strategy of inducing Drp1-dependent mitochondrial fission for cervical cancer prevention and treatment.	
INTRODUCTION			
Background	3	With an estimated 570,000 cases and 311,000 deaths in 2018 worldwide, cervical cancer is the fourth leading cancer type and the second most common gynecological malignancy among women throughout the world. With high morbidity, more than half a million women are diagnosed as new cervical cancer patients annually. Although the treatments and diagnostic tools are improved, they have side effects such as the 5-FU chemotherapy. Therefore, it is urgent to search for a cost-effective drug to treat cervical cancer.<br><br>With increasing interest of screening for active anticancer compounds from natural products, a large number of the potential target-specific anti-cancer natural compounds have been identified recently. With more than centuries of history of being used in folk medicine, *Silybum marianum* L. extract is known to have hepatocyte protective properties and it can prevent HCV infection. Therefore, it is commonly used in clinical setting. Furthermore, *Silybum marianum* L. is a dietary supplement to reduce liver toxicity since three decades ago. Silibinin (SB) is one of the chief bioactive natural polyphenolic flavonoids isolated from the fruits and seeds of *Silybum marianum* L. SB is a well-tolerated hepatic protection medicine with minimal adverse effects. Moreover, SB has shown anti-cancer activity in several clinical treatment of tumor, such as in non-small cell lung cancer patients, prostate cancer patients, and colorectal cancer patients. Given the traditional use of SB in clinic and the high anti-cancer efficacy of SB, the mechanism underlying its anti-cervical cancer activity deserves further investigation.	Introduction /1-3

		Previous study has shown that SB induces breast cancer cell apoptosis due to mitochondrial dynamic impairment. Mitochondrial dynamics contains network connection, the structure of mitochondria are with continuously fission. Fission is crucial to maintain the mitochondrial function and the cellular physiological, such as cell proliferation, energy production, and cell cycle. Cyclins and Cyclin-dependent kinases (CDKs) stringently regulate cell cycle. Cyclin B1/CDK1 complex that regulates the checkpoint of G2/M, is especially important to control the cell enter mitosis. Accumulating evidence showed that the fission-mediating GTPase dynamin-related protein 1 (Drp1) could thoroughly change the mitochondrial fission via regulating the cell cycling. Drp1 is activated by G2/M arrest and consequently induces the mitochondrial fission. Studies shown that the increasing expression of Drp1 in mitochondrial fission could induce cervical cancer cell apoptosis.	
Objectives	4	In this study, we are the first to demonstrate that SB induces G2/M cell cycle arrest in cervical cancer cells via activation of the Drp1-mediated mitochondrial fission pathway. Our research has showed that SB is a promising medicine for the treatment of cervical cancer and provides a rationale for the pivotal role of the Drp1 in G2/M cell cycle arrest.	Introduction /4
METHODS			
Ethical statement	5	All animal experiments were conducted in accordance with the animal use guidelines from the Animal Care and Use Committee of the Guangzhou Institute of Sport Science (No.GZTKSGNX-2016-1).	Materials and methods/26, 28
Study design	6	For zebrafish xenografts, the number of embryos in experimental and control groups were 50, each 10 embryos was in one well of 24-well plate. For nude mice xenografts, 6 nude mice was included in both experimental and control groups, and both same group mice were in one cage. Both zebrafish xenografts and nude mice xenografts were in randomized grouping by number all the subjects and divided	Materials and methods/26, 28

		the subjects with random selected by software into the control group and experiments.	
Experimental procedures	7	Adult zebrafish were maintained at a constant temperature of 28.5 °C with a 14/10 h light/dark cycle. Hela cells were labeled with Dil dye (2 μM, Yeasen, Shanghai, China) and approximately 300 cells were injected into yolk sac of 2 days of post fertilization embryos and incubated with indicated concentrations of SB for 24 h. The indicated concentrations treated to zebrafish were basically same to the concentration to cells. Each group was 50 embryos of 2 days post fertilization zebrafish. For each group, 10 embryos was in one well of 24-well plate. SB was mixed with egg water and added in well of 24-well plate. The cancer cell proliferation was determined by fluorescence microscopy (Olympus MVX10, Olympus, Japan).<br><br>4 week-old female BALB/c nude mice (Guangdong Laboratory Animal Center, Guangzhou, China) were housed in a specific pathogen free environment with constant temperature (22-25 ℃) and humidity (40-50 %). Hela cells (1×10^6) in the exponential growth phase were harvested and injected (100 μL per site) into the right flank of each mouse. Therapeutic experiments were started when the tumor reached about 100 mm^3. The mice were allocated to receive vehicle (control group, n=6, intragastric administration (ig)), 150 mg/kg (n=6, ig) and 300 mg/kg (n=6, ig) body weight SB and 10mg/kg 5-FU (n=6, ig) in the same volume of 0.2 mL once a days. The tumor size was measured using a slide caliper, and the tumor volume = 0.5 × length × $width^2$. Tumor volume was measured every 3 days. The mice were euthanized by cervical dislocation and tumor tissues were excised and weighed.	Materials and methods/26, 28
Experimental animals	8	Wild-type-AB line zebrafish in 2 days of post fertilization embryos were kindly provided and housed in the key laboratory of Zebrafish Modeling and Drug Screening for Human Diseases Institute at Southern Medical University (Guangzhou, China) with standard procedures of Institutional Animal Care and Use Committee (IACUC). 4	Materials and methods/26, 28

		week-old female BALB/c nude mice (Guangdong Laboratory Animal Center, Guangzhou, China) (16 ±2 g).	
House and husbandry	9	Adult zebrafish were maintained at ecosystem water (PH, 7.0-8.0, ⩾100 mg/L $CaCO_3$ and 0.25-0.75 ‰ salt) and a constant temperature of 28.5 °C with a 14/10 h light/dark cycle. And 2 days of post fertilization embryos were maintained at egg water in 37 °C incubator. 4 week-old female BALB/c nude mice were housed in a specific pathogen free environment with constant temperature (22-25 ℃) and humidity (40-50 %).	Materials and methods/26, 28
Sample size	10	For zebrafish, each group was 50 embryos of 2 days post fertilization zebrafish. For each group, 10 embryos was in one well of 24-well plate. Each group was 6 nude mice and raised in one cage.	Materials and methods/26, 28
Allocating animals to experimental groups	11	Both zebrafish xenografts and nude mice xenografts were in randomized grouping by number all the subjects and divided the subjects with random selected by software into the control group and experiments. For each group, 10 embryos was in one well of 24-well plate. SB was mixed with egg water and added in well of 24-well plate. The mice were allocated to receive vehicle (control group, n=6, intragastric administration (ig)), 150 mg/kg (n=6, ig) and 300 mg/kg (n=6, ig) body weight SB and 10mg/kg 5-FU (n=6, ig) in the same volume of 0.2 mL once a days. The tumor size was measured using a slide caliper, and the tumor volume = 0.5 × length × width^2. Tumor volume was measured every 3 days.	Materials and methods/26, 28
Experimental outcomes	12	In zebrafish xenografts, the cancer cell proliferation was determined by fluorescence intensity in yolk sac. In nude mice xenografts, the tumor size was ≤ 600 mm^3.	Materials and methods/26, 28
Statistical methods	13	All data are expressed as the means ± SDs and analyzed by SPSS 20.0 (IBM, Armonk, USA). Tukey's test was used for multiple comparison. The values were considered statistically significant when $P < 0.05$.	

RESULTS			
Baseline data	14	For each experimental group, no prior treatment or testing, and the weight of nude mice was 16 ±2 g, the size of embryos was same.	Results/ supplement figure 3
Numbers analysed	15	In zebrafish xenografts, the number of embryos in each group in each analysis was 50/50. In nude mice xenografts, the number of mice in each group in each analysis was 6/6.	Results/15
Outcomes and estimation	16	For zebrafish xenografts fluorescence intensity, the 95 % confidence interval in each group, compared with control group, was 4.640 to 46.99, 19.34 to 61.69, 56.43 to 98.78 treated with SB separately, and treated with 5-FU was 36.25 to 78.60. For nude mice xenografts tumor weight, the 95 % confidence interval in each group was 0.6360 to 1.455, 1.453 to 2.272 separately in SB treatment, and 1.115 to 1.934 in 5-FU treatment.	Results/15
Adverse events	17	There had no important adverse events in each experimental group, and the volume of tumor was measured at each three day to avoiding the tumor volume was too big to affect the status of nude mice.	Results/15
DISCUSSION			
Interpretation/scientific implications	18	Our study confirmed our hypotheses that SB could inhibit the growth of cervical cancer tumor in vivo, and suggests a novel novel therapeutic strategy for cervical cancer prevention and management. In this animal model, we choose unconscious zebrafish embryo combined with nude mice as xenograft models to reduce using of nude mice number, using inhalation anesthesia to relieve pain and distress caused to nude mice in experiment. The potential source of bias and limitations of the zebrafish embryo would be that sex of embryo couldn't be sure, but this would not affect the results of experiment.	Discussion/ 7
Generalisability/ translation	19	In cancer research, cell transplantation approaches have been used to detect molecular pathways involved in tumor growth, metastasis and anti-tumor therapies. Among these	Discussion/ 7

		approaches, xenograft human cancer models can be readily engrafted into immune-deficient mice. Zebrafish, a vertebrate model system, becomes an important new cancer model which complements the functions of traditional xenograft mouse model and also has the capabilities to capture the heterogeneous and evolving complexity of cancer *in vivo*. We found that in both zebrafish tumor model and xenograft mouse model, SB inhibited Hela cells growth, which suggest the possibility of using SB as a new clinical therapeutics for cancer treatment.	
Funding	20	This work was supported by the Key Project of National Natural Science Foundation of China (No. 81830117), the National Science Foundation of China (No. 81673840, 81873205), the Natural Science Foundation of Guangdong Province, China (No. 2017A030313791), and the Science & Technical Plan of Guangzhou, Guangdong, China (No. 201804010118).	Funding/1
