# Supplementary material for: Silibinin Induces G2/M Cell Cycle Arrest by Activating Drp1-Dependent Mitochondrial Fission in Cervical Cancer
Source: Front Pharmacol. 2020 Mar 12;11:271. doi: 10.3389/fphar.2020.00271 (PMC7080994; doi:10.3389/fphar.2020.00271)
Supplement: Supplementary file 2 [file Data_Sheet_2.DOCX]

**Supplymentary Materials: Silibinin induces G2/M cell cycle arrest by activating Drp1-dependent mitochondrial fission in cervical cancer**

**Supplementary material**

The following primers were used for q-PCR. *β-actin*, forward 5′-GAAATCGTGCGTGACATTAAAG-3′ and reverse 5′-ATCGGAACCGCTCATTG-3′; mtDNA, forward 5′-CGAAAGGACAAGAGAAATAAGG-3′ and reverse 5′-CTGTAAAGTTTTAAGTTTTATGCG-3′; *drp1*, forward 5′-GGAGACTCATCTTTGGTGAAGAG3′ and reverse 5′-AAGGAGCCAGTCAAATTATTGC-3′; *fis1*, forward 5′-GTCCAAGAGCACGCAGTTTG-3′ and 5′-ATGCCTTTACGGATGTCATCATT-3′; *opa1*, forward 5′-TGTGAGGTCTGCCAGTCTTTA-3′ and reverse 5′-TGTCCTTAATTGGGGTCGTTG-3′; *mfn1*, forward 5′-TGGCTAAGAAGGCGATTACTGC-3′ and 5′-TCTCCGAGATAGCACCTCACC-3′; *mfn2*, forward 5′-CTCTCGATGCAACTCTATCGTC-3′ and 5′-TCCTGTACGTGTCTTCAAGGAA-3′.

The following primers were used for Drp1-siRNA. Drp1-siRNA1, 5’-CAGGAGCCAGCTAGATATTAA-3’; Drp1-siRNA2, 5’-GCCAGCUAGAUAUUAACAACAAGAA-3’; Drp1-siRNA3, 5’-GGAACGCAGAGCAGCGGAAAGAGCT-3’.

**Supplementary figure**


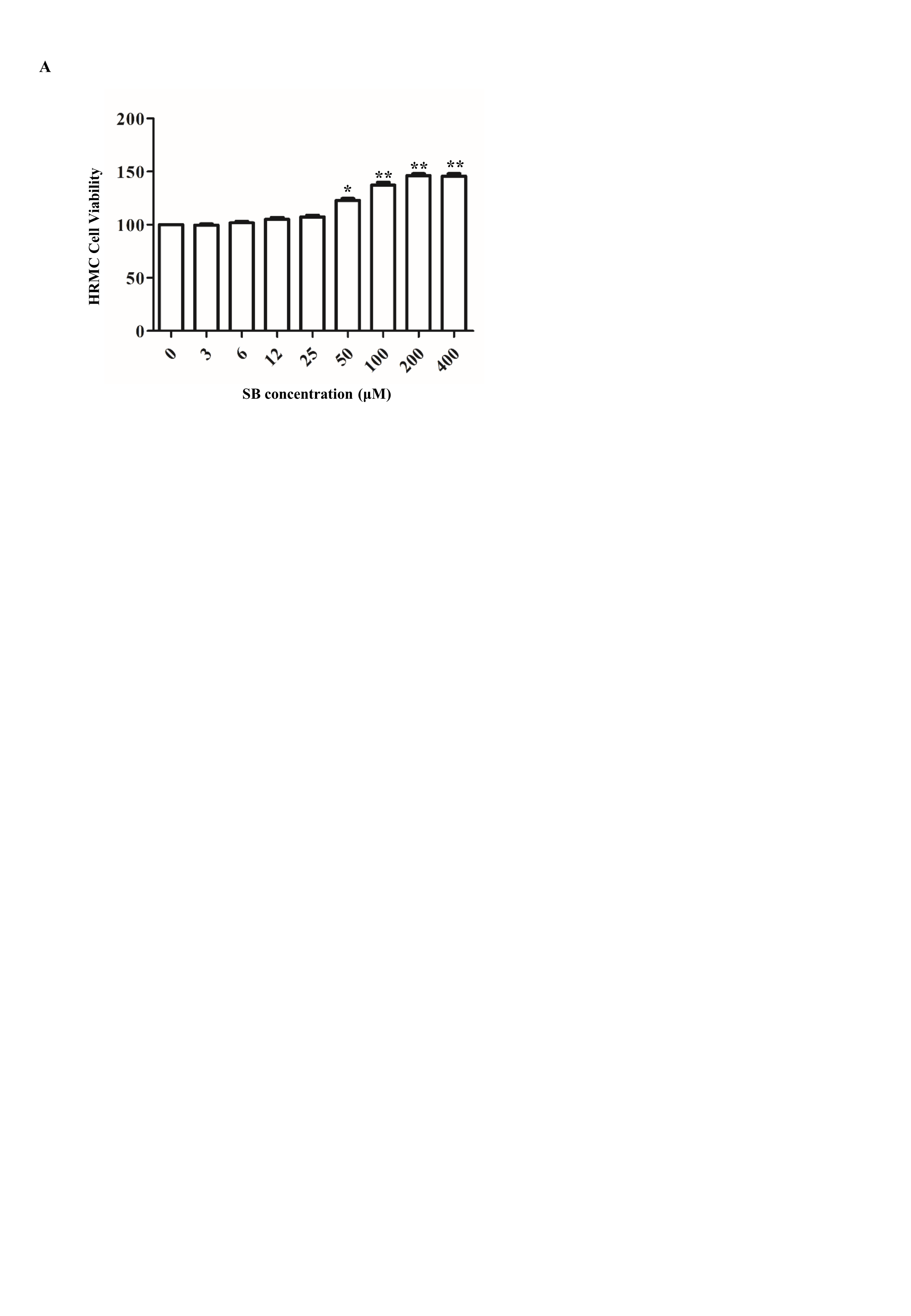


Fig S1. Cells viability analysis of SB in normal Human Renal Mesangial Cells (HRMC).

Cells were exposed to silibinin (SB) at different concentrations for 24 h, and cell viability was measured by MTT assays. Values (mean ± SDs) were obtained from at least three independent experiments. *P < 0.05, **P < 0.01 and ***P < 0.001 by one-way ANOVA with Tukey’s test.

.


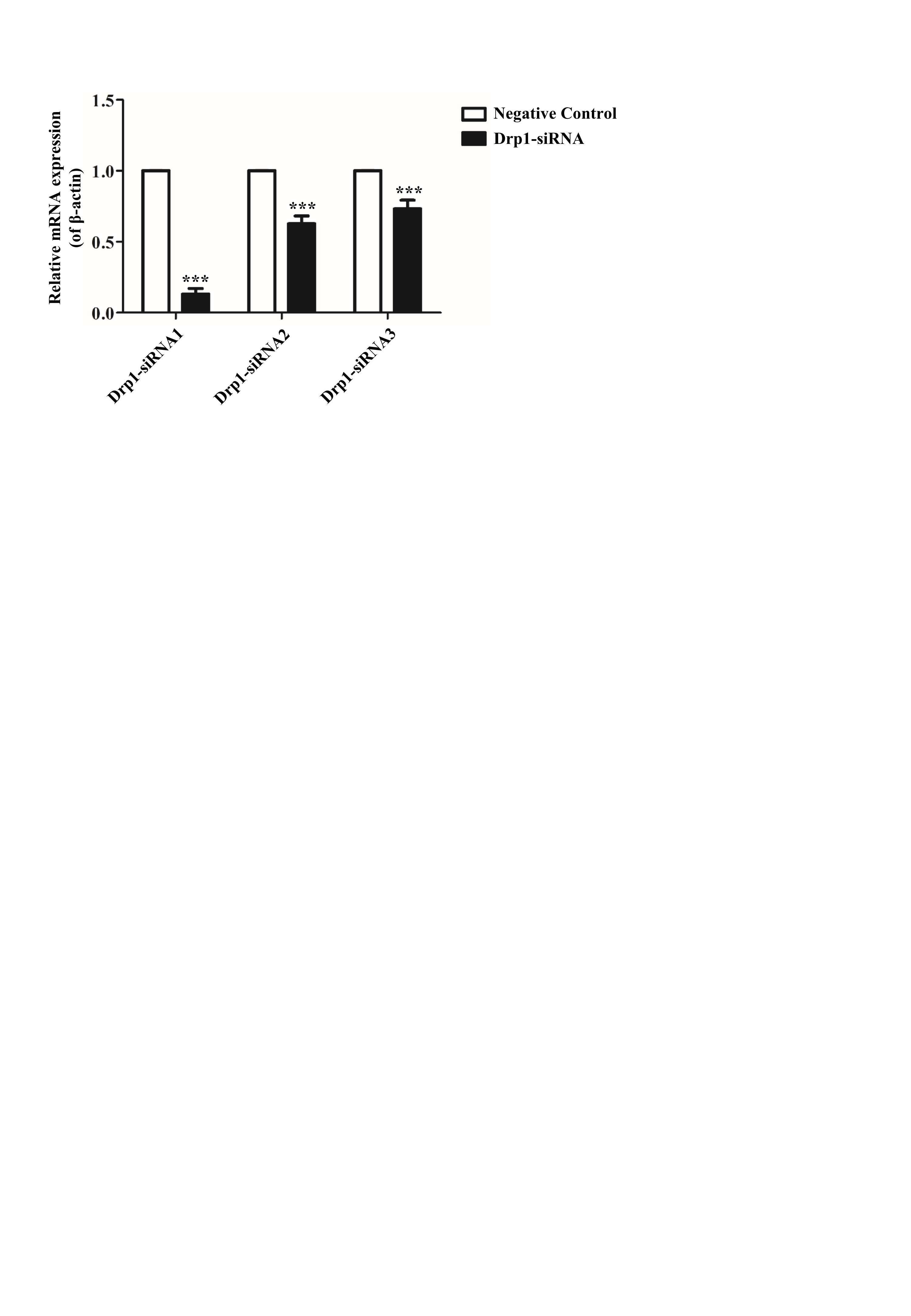


Fig S2. qPCR analysis of Drp1-siRNA in Hela cells. Hela cells were transfected with Drp1-siRNA (100 nM) or negative control for 24 h.


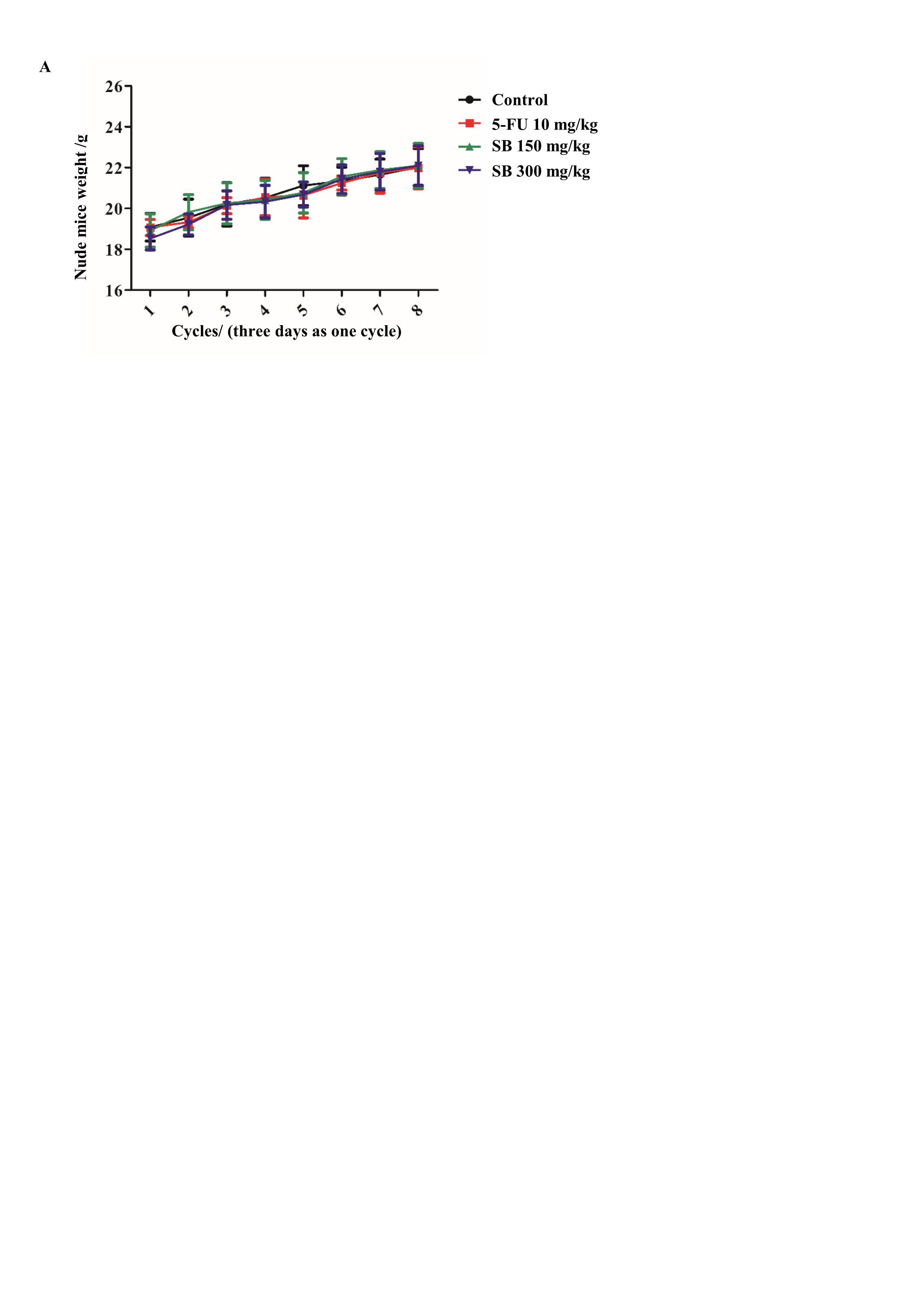


Fig S3. SB doesn’t affect the weight of nude mice in xenograft mouse model. Values (mean ± SDs) were obtained from the independent experiments. *P < 0.05, **P < 0.01, and ***P < 0.001, versus control group by one-way ANOVA with Tukey’s test.
